# Assessing variational likelihood estimation in network formation models

**DOI:** 10.1371/journal.pone.0358535

**Published:** 2026-10-06

**Authors:** Joseph Resch, Mark S. Handcock

**Affiliations:** Department of Statistics & Data Science, University of California - Los Angeles, Los Angeles, California, United States of America; National Research Council: Consiglio Nazionale delle Ricerche, ITALY

## Abstract

Networks are often a useful way to represent complex social and economic systems. Stochastic models for such networks should parsimoniously reflect the complexity while being both interpretable and tractable. The class of Exponential-family Random Graph Models (ERGMs) is very expressive and flexible in its representation of complex phenomena. However, the estimation and fitting of realistic ERGMs are hampered by the computational challenges of likelihood-based inference. Recently, a deterministic method based on a variational mean-field approximation was proposed that is much less expensive to compute than the standard simulation-based method. In this work, we show that the proposed method performs poorly for realistic models. We show that established alternatives, including MCMC-MLE, MPLE, sampled MPLE, and tapered ERGM-based inference, achieve superior accuracy and computational performance in the situations that the mean-field approximation was designed for.

## 1. Introduction

There has been increasing interest in models for complex stochastic phenomena. Exponential-family models are able to represent complex structure in an interpretable way [1,2]. This is particularly true of stochastic models for networks, where the nodes represent actors and the edges relational ties between those actors. The configuration of these ties and their dependence on attributes of the nodes influence how processes unfold on networks. Exponential-family random graph models (ERGMs) offer a flexible framework for representing these dependencies [3–5]. There is a substantial literature on these network models and their applications across disciplines [5–7].

### 1.1 Exponential-family random graph models

ERGMs are a family of random graph models that have received extensive attention in the statistical literature and are widely used in applications [8,9]. A network may be represented as graph *G* = (*V*,*E*) using node set *V* and edge set *E*, where nodes are the actors of interest and edges define the relational tie. The edges can be binary or valued/weighted and directed or undirected [10]. For example, edges can be undirected binary values indicating whether a relationship exists between two nodes, or not. In many situations the number of nodes is fixed and known (*n* = |*V*|). In this case we have as many as 2(n2) possible graphs. The heterogeneous nodes, indexed by i=1,...,n, often have an associated vector, $x_{i}$, of covariates representing attributes such as age, income, and location.

The model represents an observed network as a realization $y\in G_{n}$ of random network *Y*, where $G_{n}$ is the set of all possible networks *Y* with *n* nodes. *Y* is often represented as an n×n matrix of relational variables with $(i,j{)}^{th}$ element the value of the edge from node *i* to node *j*. In the binary undirected case, realization $y_{i,j}$ takes values from {0,1} where $y_{i,j}=y_{j,i}$ for 1≤i,j≤n. Typically, any self ties of the nodes are not represented so that $y_{i,i}$ is undefined for all i∈[1,n]. For notational convenience, dependence on the covariates $x_{1},...,x_{n}$ is suppressed. The probability of observing *Y* = *y* is given by:


$$\begin{array}{c} p_{\theta }(Y=y)=\frac{\exp(\theta \cdot Z(y))}{c(\theta ,G_{n})}\;y\in G_{n}. \end{array}$$ (1)

ERGMs are defined by the choice of network statistics, *Z*(*y*), a d−dimensional vector-valued function that specifies a set of sufficient statistics. The associated parameter vector is $\theta \in {\mathbb{R}}^{d}$, and $c(\theta ,G_{n})$ denotes the normalizing constant. A basic statistic is the total number of edges (‘Edge’, $\sum_{i=1,j=1}^{n,n}y_{i,j}$). Most models also include statistics defined over triads (sub-graphs of triplets of nodes), especially measures of transitivity, the propensity for ties between pairs of nodes who are tied to a common third node. Examples are the clustering of edges (‘Two-Star’, $\sum_{i=1,j=1,k=1}^{n,n,n}y_{i,j}y_{i,k}$) and transitivity (‘Triangle’, $\sum_{i=1,j=1,k=1}^{n,n,n}y_{i,j}y_{i,k}y_{j,k}$). If there is a nodal attribute $x_{i}$ then one can measure homophily of edges on it via (‘Homophily’, $\sum_{i=1,j=1}^{n,n}𝕀(x_{i}=x_{j})y_{i,j})$. There are a multitude of choices for these statistics (See Morris et al. [11] for a list). In this way, ERGMs constitute a family of models across different choices of the network statistics. Regardless of which network statistics are used, an ERGM is the unique distribution of maximum entropy among all distributions on $G_{n}$ whose expected sufficient statistics are fixed and satisfy the moment constraint ${𝔼}_{\theta }[Z(y)]=\mu$, where *Z*(*y*) is the d−dimensional vector of network statistics and $\mu \in {\mathbb{R}}^{d}$ is a specified vector of mean values [12].

### 1.2 Likelihood-based inference for ERGM

For undirected binary networks, the normalizing constant $c(\theta ,G_{n})=\sum_{y\in G_{n}}\exp(\theta \cdot Z(y))$ accounts for all 2(n2) possible graphs, which makes the summation computationally intractable even for networks with few nodes. The calculation of this term is crucial to likelihood-based inference. Markov chain Monte Carlo (MCMC) simulation is a popular approach to approximate the likelihood by simulating many networks and using them to estimate the intractable normalizing constant [9,13]. In another approach called maximum pseudo-likelihood estimation (MPLE), a pseudo-likelihood is used in place of the true likelihood [14]. Whereas the exact properties of the MPLE vary by case, using MCMC to obtain an approximate MLE (MCMC-MLE) has become the workhorse in this area with much research and practical knowledge [15–17]. This is enhanced by the availability of sophisticated user-friendly open-source software for MCMC-based inference [9]. However, in most cases MPLE’s computational simplicity may outweigh the benefits of MCMC-MLE [14].

Over time, substantial efforts have been made to improve likelihood-based inference for ERGM [15–17]. In that effort, Mele and Zhu [18] proposed a method to approximate ERGM likelihood using a variational mean-field and demonstrated its effectiveness through a microeconomic application. Their method minimizes the Kullback–Leibler (KL) divergence between a tractable approximating distribution and the true ERGM likelihood by maximizing a lower bound on the intractable normalizing constant. Unlike MCMC approximation of the intractable likelihood, maximum mean-field variational likelihood estimates (MFVLE) are deterministic.

A key feature of the mean-field approximation is that it replaces the full ERGM, with its complex dependence among ties, by a model with conditionally independent ties whose parameters are chosen to be as close as possible to the ERGM under the KL criterion. This factorization results in a variational problem whose solution provides an approximate log-likelihood without requiring any simulation. Mele and Zhu [18] show that the approximation error of this mean-field log-constant converges to zero as the number of nodes increases, using tools from nonlinear large deviations theory. Consequently, the mean-field log-likelihood uniformly approximates the true ERGM log-likelihood in large networks, and the MFVLE converges to the MLE when the underlying ERGM likelihood is well behaved. They also provide explicit bounds on the approximation error, and their studies suggest that, even for moderately sized networks, the approximation performs better than these bounds indicate.

These results suggest that the variational mean-field approach provides a computationally efficient and theoretically grounded alternative to standard simulation-based ERGM estimation. Our results below suggest that this conclusion does not extend to the tie-dependent models that motivate many ERGM applications, for which the approximation is both inaccurate and more expensive to compute than established methods.

### 1.3 Outline of the paper

We show that the finite-network estimation experiments in Mele and Zhu [18] rely on initialization at the true parameter values, a design choice that is unavailable in applications and that materially changes the apparent performance of MFVLE. We address this and replicate the study on 1,000 sampled networks, then show that the empirical performance of the method is poor and dramatically worse than the MCMC-MLE. We replace initialization at the true parameter values with practically attainable starting values, primarily the MPLE, while focusing on how well the mean-field likelihood approximation functions under certain levels of network transitivity. A useful tool here comes from van Duijn et al. [14], where transitivity in ERGM natural parameters can be systematically adjusted by way of one-to-one mapped mean-value parameterization. In turn, manually increased transitivity allows us to stress test the estimation methods under more complex network structures. Our findings are accompanied by conceptual reasoning on why the mean-field method will be poor in circumstances where it is most needed.

The paper is organized as follows. Section 2 reviews ERGM specifications, likelihood-based inference methods, and other conceptual issues related to the mean-field likelihood approximation for ERGM. Section 3 discusses the simulation study framework and compares the mean-field variational method against current methods. Section 4 draws conclusions on the contents of this paper.

## 2. Theoretical considerations

### 2.1 Realism of the proposed ERGM class

The choice of sufficient statistics is critical in an ERGM specification. Here we consider the choice of Mele and Zhu [18]. They used a microeconomics model to explore best-response equilibrium dynamics in potential games by measuring players’ payoffs. Real-world networks, such as those in social science and biology, tend to have tie dependence that causes clustering of the edges and transitivity. The microeconomics model reflects this by including Two-star and Triangle terms.

One aspect of model specification that is important to take into consideration is that certain combinations of terms can result in the model not placing sufficient probability mass on realistic graphs and networks. This is called the model degeneracy problem [19–22]. For example, using a Triangle statistic as a measure of transitivity will lead to degeneracy in many cases. This is because a single edge can form the basis for a large number of triangles, so using the unweighted count of the number of triangles can create strong positive feedback in the sufficient statistics. An ERGM with a positive coefficient on a Triangle term may therefore place substantial probability mass on unrealistic regions of the sample space, such as nearly empty or nearly complete graphs, or on extreme regions of the convex support of the network statistics.

Mele and Zhu attempt to reduce this degeneracy by scaling the coefficients of the Two-star and Triangle terms [23,24], but this also reduces the realism of the model. We return to the problem of fitting realistic, transitive models in Sections 3.4–3.6, where we adopt a tapered ERGM that retains the Triangle term while controlling degeneracy.

### 2.2 Theoretical analysis of MFVLE

The core idea of variational approximation is to use a variational representation of the log-normalizing constant. For an ERGM, this representation can be written as a supremum over the set of all probability mass functions on $G_{n}$ [28, Section 2C]. This exact variational problem is as difficult as the original likelihood problem, because it ranges over all probability distributions on the network sample space. Mele and Zhu therefore restrict the variational class to the set of tie-independent probability mass functions. In this restricted class, the approximating distribution factorizes over dyads, and the variational problem has a much simpler computational form.

This restriction is not merely a computational device. It changes the dependence structure of the model being used to approximate the likelihood. The ERGM likelihood assigns probability to entire networks and, through statistics such as Two-stars and Triangles, represents endogenous dependence among dyads. The mean-field approximation replaces this joint distribution by a product distribution over dyads. Thus the approximation is structurally closest to the ERGM when the fitted model is close to dyad independence, and it is least faithful when the likelihood is shaped by the dependence terms that motivate the use of ERGMs in the first place. This observation is central to our assessment of MFVLE: the relevant question is not whether mean-field approximations can be accurate in favorable weak-dependence regimes, but whether this particular dyad-factorized approximation remains reliable for finite networks with realistic transitivity and other forms of endogenous tie dependence.

The related theoretical literature gives reason to expect such failures. In closely related Ising models, mean-field approximations can be accurate in highly averaged settings such as complete graphs [25], but can perform poorly in sparse settings [26]. This distinction is important for network models, because empirical social and economic networks are often sparse or semi-sparse, and their dependence is usually local rather than complete-graph-like. Ricci-Tersenghi [27] shows that mean-field methods can perform poorly when dependence is strong. Lokhov et al. [28] similarly find poor performance for strongly coupled systems and degenerate regions of the parameter space, and contrast this behavior with MPLE and other more robust methods. These results suggest that the performance of MFVLE should depend strongly on where the true or fitted model lies in the parameter space. In particular, strong coupling, degeneracy, and transitivity are not peripheral cases for ERGMs; they are precisely the features often introduced to make the model substantively useful.

Related work on ERGM-specific mean-field approximations points in the same direction. Biondini et al. [29] consider a mean-field approximation for ERGMs based on *k*-star statistics. Under their asymptotic scheme, the approximation can represent macroscopic features, such as edge density, while failing to represent microscopic dependence, such as dyadic dependence. This distinction is directly relevant here. A mean-field approximation may reproduce some low-dimensional aggregate features of a network while still misrepresenting the dependence structure induced by Two-star and Triangle terms. Consequently, good approximation of an edge density or of a scaled log-normalizing constant does not by itself imply accurate estimation of parameters governing endogenous dependence.

The same point can be expressed through the geometry of the exponential family. The log-partition function is convex in the natural parameter, and the log-likelihood


$$\begin{array}{c} \ell (\theta ;y)=\theta^{⊤}Z(y)-\log c(\theta ,G_{n}) \end{array}$$

is concave, with curvature determined by $\nabla^{2}\ell (\theta ;y)=-{\mathrm{Var}}_{\theta }[Z(Y)].$ Thus the shape of the likelihood is governed by the covariance structure of the sufficient statistics. For ERGMs with transitivity terms, this covariance structure is induced by higher-order dependence among overlapping dyads. A dyad-factorized variational family removes this dyadic dependence in the approximating distribution. Although nonlinear statistics such as Two-stars and Triangles can still be evaluated under the product distribution, the approximation cannot generally reproduce the dependence-induced curvature of the true ERGM likelihood. In poorly conditioned regions of the convex support, or in regions near faces of the support associated with degeneracy, the KL projection onto the mean-field family can therefore lie far from the true model. The resulting approximate likelihood may have a substantially different shape from the true likelihood, leading to biased point estimates and misleading measures of uncertainty.

This concern is amplified by the direction of the variational approximation. MFVLE minimizes $KL(q\|p_{\theta })$ over a restricted class of product distributions *q*. In many multimodal or strongly dependent problems, this direction of Kullback–Leibler divergence has a mode-seeking tendency: it can favor an approximating distribution that concentrates on one part of the probability mass rather than representing the full dispersion or multimodality of the target distribution. Such behavior is especially problematic in ERGM regions with strong dependence, where the distribution over networks may place substantial mass near separated regions of the sample space or near the boundary of the convex support. This provides a theoretical mechanism for the empirical failures examined below.

There is also a distinction between approximation of the log-normalizing constant and estimation of the model parameters. The main result of Mele and Zhu, Proposition 1, shows that the mean-field approximation to the scaled log-likelihood converges to the ERGM scaled log-likelihood as the number of nodes, *n*, increases, under assumptions controlling the growth of the parameters $\theta_{n}=(\alpha_{n},\beta_{n},\gamma_{n}).$ This result addresses an approximation question: for a specified parameter sequence and asymptotic regime, how close is the mean-field approximation to the true log-normalizing constant or scaled log-likelihood? The inferential question is different: when the approximation is maximized for an observed finite network, how close is the MFVLE to the MLE? Uniform approximation of likelihood values does not by itself guarantee accurate approximation of the maximizer unless additional conditions on the shape of the likelihood hold, such as strict or strong concavity and appropriate identification. These conditions are precisely the ones that may be weak or fail in degenerate ERGM regions.

The asymptotic regime considered by Mele and Zhu is also close in spirit to dense-graph asymptotics, in which sufficient statistics scale as *n*^2^ and the network does not become sparse as *n* increases [23,24]. Many empirical social and economic networks are sparse or semi-sparse, with edge counts growing sub-quadratically in *n*. Moreover, the number of nodes is not a tuning parameter under the researcher’s control. It is a structural feature of the phenomenon being modeled. Networks with different sizes often correspond to different social or economic systems, and there is no general reason to assume that their natural parameters follow a convenient asymptotic sequence. For example, consider social relations in a school classroom with *n* = 3 students in it compared to a classroom with *n* = 100 students. The social dynamics are quite different and the $\theta_{n}$ should be as well. For a broader discussion of this issue, see Krivitsky et al. [30].

This distinction also clarifies why the analogy between MFVLE and MCMC-MLE is limited. Both methods approximate the log-normalizing constant, but the source of approximation is fundamentally different. In MCMC-MLE, the simulation size is chosen by the researcher and can, at least in principle, be increased to reduce Monte Carlo error. In the mean-field asymptotic argument, by contrast, the quantity that grows is the number of nodes in the observed network. For any particular application, *n* is fixed by the phenomenon under study. Increasing *n* is therefore not an available computational refinement in the same sense as increasing the length of an MCMC run. Thus the fact that the mean-field approximation improves under a particular increasing-*n* regime does not imply that it is accurate for the fixed finite networks encountered in applications.

The potential-game specification used by Mele and Zhu further illustrates the issue. The final term in their utility specification counts the number of common friends, which corresponds to a Triangle term in the ERGM. Positive coefficients on Triangle terms are well known to produce degeneracy over wide regions of the parameter space [19]. In the scaled specification studied here, choosing the Triangle coefficient small enough to avoid degeneracy can place the model close to dyad independence, a regime in which MPLE is expected to be effective.

These considerations motivate the simulation design below. We compare MFVLE to MCMC-MLE and MPLE in the original model class studied by Mele and Zhu, but we do so under practically attainable initialization rather than initialization at the true parameter value. We also examine performance in both the natural and mean-value parameterizations. The mean-value parameterization allows us to increase transitivity directly and thereby assess estimation accuracy in the regions where dyad-factorized mean-field approximations are expected to be most fragile. The purpose of the simulation study is therefore not simply to ask whether MFVLE can work in weak-dependence examples, but to determine whether it remains reliable in the finite-network, transitive regimes where likelihood approximation is most needed.

### 2.3 A framework for the comparison of estimation methods

A framework for the comparison of estimators for ERGM was developed by van Duijn et al. [14]. Here we review the core elements of that framework.

It is well known that MCMC-MLE converges to the MLE as the sampling increases [13] and can provide useful results with enough iterations. However, convergence is often not practically available due to degeneracy and MCMC variability [19,21]. Maximum pseudo-likelihood estimation is a popular alternative choice, and is used in many applications for its advantage in computational time compared to using MCMC. While both are approximations to the exact MLE, the error of the MCMC-MLE can be reduced by additional computation.

Handcock [19] proposed the consideration of the alternative *mean-value parameterization* ERGM, μ(θ), that has the one-to-one mapping with natural parameters θ characterized by:


$$\begin{array}{c} \mu (\theta )={𝔼}_{\theta }[Z(y)]. \end{array}$$ (2)

The mapping is strictly increasing in the sense that:


$$\begin{array}{c} {\left(\theta_{a}-\theta_{b}\right)}^{T}\left(\mu \left(\theta_{a}\right)-\mu \left(\theta_{b}\right)\right)\geq 0 \end{array}$$ (3)

with equality only if $P_{\theta_{a}}(Y=y)=P_{\theta_{b}}(Y=y)\;\forall y$. The mapping is also injective in the sense that


$$\begin{array}{c} \mu \left(\theta_{a}\right)=\mu \left(\theta_{b}\right)\to P_{\theta_{a}}(Y=y)=P_{\theta_{b}}(Y=y)\;\forall y. \end{array}$$ (4)

The mean value parameterization has the main advantage of interpretability due to its direct translation to the network statistics chosen for the model. van Duijn et al. [14] adopted the mean-value parameterization to examine performance of MCMC-MLE and MPLE under different transitivity scenarios. Their results show the MPLE performs as well as the MCMC-MLE when there is little transitivity/tie-dependence in the model. However, where there is realistic transitivity/tie-dependence the MCMC-MLE outperformed MPLE. It is worth noting that the performance gains of MCMC-MLE over MPLE are most pronounced for parameters capturing endogenous tie dependence, whereas the improvement for nodal and dyadic covariates such as Homophily is comparatively modest.

An increase in transitivity cannot be easily induced in the natural parameterization, as a direct change in a natural parameter leads to complex changes in the distributions of the network statistics due to interactions. However, transitivity can be added by increasing the corresponding mean-value parameter. The mean-value parameters with the added transitivity can then be inversely mapped to the vector of natural parameters (See van Duijn et al. [14] for details).

As model transitivity is increased by way of the mean-value parameterization, the superior performance of MCMC-MLE becomes more noticeable as the bias of the MPLE for both the natural parameters and mean-value parameters increases. Despite this inferior performance in realistic settings, the MPLE serves as a useful initialization for more complex methods, such as the MCMC-MLE. In fact, it is the default initialization for the state-of-the-art MCMC-MLE in the ergm R package [9,31]. One factor to keep in mind is the likelihood of degeneracy for the estimates (that is, high proportion of mass placed on the boundary of the convex hull of network statistics). The degeneracy of a model fit can be assessed by the R package ergm [9,31].

## 3. Study design and simulation results

In this section we use the framework of van Duijn et al. [14] to design a simulation study to assess the accuracy of the MFVLE compared to the MCMC-MLE and MPLE.

We first replicate the Monte Carlo simulation study of Mele and Zhu [18], Section 4.2. A key feature of their study was initiating the optimization at the true values. They find that the estimates do not vary much from the start point, and interpret this as accuracy. We replicate their study but start from the MPLE, which is by far the most common initialization point for estimates [15]. We try larger network sizes to see if that improves the MFVLE, and then show that the parameter values they use are close to a tie-independent model. Such parameter values are rarely realistic and so we modify the parameter values to produce a similar model with more realistic transitivity (i.e., an increased level). We then repeat the study for models with this more realistic transitivity.

### 3.1 Re-evaluating the Monte Carlo simulation study of Mele and Zhu [18]

The same ERGM specification as in Mele and Zhu [18] – in which the chosen sufficient statistics are Edges, Homophily, Two-stars, and Triangles – is used. The predetermined true parameters corresponding to the models are dependent on network size.

We note that there is a defect in their simulation study. As stated at the end of their Section 4B, “To be sure that our results do not depend on the initialization of the parameters, we start each estimator at the true parameter value.” Mele and Zhu [18] set the initialization of model parameters in the algorithm for both MCMC-MLE and MFVLE to be the true parameters that are used to simulate networks. However, these true parameters are not known in practice. Moreover, their use of the true values obscured the fact that the algorithm did not move the parameter values toward the true values, but only started there. Thus the reported experiment does not evaluate how MFVLE behaves from attainable starting values, and its apparent accuracy cannot be interpreted as evidence of reliable estimation performance.

In contrast, we use the MPLE, the current standard method, as the initial values to validate our claim. To ensure that the mean-field approximation does not converge to a suboptimal local solution, a known risk with variational methods, the MPLE initial points were perturbed with random noise, effectively restarting the variational approximation procedure over the large number of simulated networks. We also verified that initializing at the true parameter values reproduces the results of Mele and Zhu [18] deterministically. Other initial estimates, including zero vectors and random Gaussian starting points, produced similar results for both the MCMC-MLE and MFVLE. The default pipeline in the ergm package uses the MPLE to initialize the Stepping algorithm of Hummel et al. [16], which in turn seeds the MCMC-MLE. We additionally applied the same Stepping-based initialization to the mean-field method, and this did not noticeably change the mean-field estimates.

To generate artificial networks in practice, a Metropolis-Hastings network sampler is used with the initialization being an iteratively simulated network using the true parameters with attributes drawn from a Bernoulli distribution. Here, 1,000 networks are simulated for model fitting while 10,000 sets of summary statistics are simulated for mapping of mean-value parameterization. This was done using the ergm package to be consistent with Mele and Zhu [18] (although it was mentioned and used, the package was not cited by them).

### 3.2 Original study with adjusted initialization

Table 1 immediately shows the central failure of MFVLE in mean-value parameterization. For *n* = 100, the true expected number of triangles is 117.74 and the MCMC-MLE estimate is 117.76, whereas the MFVLE estimate is 6623.04. The same pattern appears for Two-stars: the true value is 3091.85, the MCMC-MLE estimate is 3091.37, and the MFVLE estimate is 33030.41. These are not small numerical discrepancies, but large failures to reproduce the expected dependence statistics of the fitted model.

**Table 1:** Original study with the initial values for both MCMC-MLE and MFVLE as MPLE. The model has true natural parameters at θ=(−4,2,1/n,1/n).

n = 50	Edge	Homophily	Two-Star	Triangle	Degeneracy
True Natural parameters	−4.000	2.000	0.020	0.020	–
MCMC-MLE Natural	−3.851	2.050	−0.006	−0.042	4.6%
MFVLE Natural	−36.532	−14.502	−0.980	−2.202	9.0%
MPLE Natural	−3.868	2.044	−0.005	−0.020	0.0%
True Mean-Value parameters	95.80	82.79	363.76	13.96	–
MCMC-MLE Mean-Value	95.99	82.93	365.08	14.02	–
MFVLE Mean-Value	263.05	163.04	5645.46	1189.43	–
MPLE Mean-Value	139.89	104.40	2561.62	743.19	–
**n = 100**	**Edge**	**Homophily**	**Two-Star**	**Triangle**	**Degeneracy**
True Natural parameters	−4.000	2.000	0.010	0.010	–
MCMC-MLE Natural	−3.916	2.005	0.004	0.005	0.4%
MFVLE Natural	−7.386	−24.215	−0.253	−0.212	2.5%
MPLE Natural	−3.915	2.003	0.004	0.008	0.0%
True mean-value parameters	393.12	340.94	3091.85	117.74	–
MCMC-MLE Mean-Value	393.07	340.86	3091.37	117.76	–
MFVLE Mean-Value	910.53	621.64	33030.41	6623.04	–
MPLE Mean-Value	416.69	352.13	5547.74	936.87	–

With the initialization adjustment applied, Table 1 partially replicates the motivating comparison for $\theta =\{-4,2,\frac{1}{n},\frac{1}{n}\}$ for *n* = {50, 100} (See Mele and Zhu [18] for details). Note the parameters displayed by the authors $\left\{\tilde{\alpha_{1}},\tilde{\alpha_{2}},\beta ,\gamma \}=\{-2,1,1,1\right\}$ are scaled by $\left\{2,2,\frac{1}{n},\frac{1}{n}\right\}$, as the authors make explicit in their provided MFVLE code implementation. We produce the MCMC-MLE using R package ergm directly from the log-likelihood function rather than the stochastic approximation [32] chosen by the authors. A burn-in of 100,000 iterations and interval of 1,000 iterations are used to be consistent with the original comparison. The MFVLE procedure is directly implemented using the R package mfergm made available on GitHub by the authors [33]. To ensure proper mapping from natural parameters to mean-value parameters, 10,000 networks are simulated to take the expected counts of sufficient statistics.

Table 1 displays the following values: true natural parameters, MCMC-MLE natural parameter estimates, MFVLE natural parameter estimates, MPLE natural parameters used to initialize both methods, and the one-to-one mean-value parameter estimates mapped from the natural parameters for truth, MCMC-MLE, MFVLE, and MPLE. For these results, the mean is used for both natural and mean-value parameterization. The right-side column shows the proportion of the 1,000 fits whose estimated parameters imply a degenerate model under each method. Degeneracy is a property of the model rather than of the estimation method, but because the methods land in different regions of the parameter space, they differ in how often their estimates imply degeneracy. A low proportion indicates that a method’s estimates fall largely in non-degenerate regions. Note that the true MLE is exactly equal to the true values, so that the error in the MCMC-MLE is a computational error. This is partially induced by the degeneracy of the model [19]. While MPLE resembles MCMC-MLE in natural parameterization, the difference between the two methods is stark in the expected count of transitivity terms. The bias in MPLE mean-value parameter estimates is induced by a set of samples with extreme values, similar to the results of van Duijn et al. [14].

Table 2 provides root mean squared error (RMSE) and mean absolute deviation (MAD) for the results in Table 1. The variability of the MFVLE results stands out greatly compared to the variability of the MCMC-MLE in both natural and mean-value parameterization. The large variability in MFVLE results was not observed by Mele and Zhu [18]; in fact, the authors reported lower mean absolute deviation in the results of MFVLE compared to that of MCMC-MLE. Although we were able to deterministically reproduce the authors’ results when initializing at the true parameter values, these findings substantiate our concerns. That is, MFVLE appears to have performed well in Mele and Zhu [18] primarily because the true parameter values were used as the initial values, which is the only modification our study has made relative to the original study.

**Table 2:** Summary measurements RMSE and MAD for *n* = 100 results in Table 1.

n = 100		Edge	Homophily	Two-Star	Triangle
MCMC-MLE Natural	RMSE	0.335	0.157	0.020	0.101
	MAD	0.273	0.130	0.016	0.082
MFVLE Natural	RMSE	3.904	6.986	0.060	0.137
	MAD	2.655	4.436	0.045	0.108
MPLE Natural	RMSE	0.338	0.156	0.021	0.103
	MAD	0.278	0.130	0.017	0.084
MCMC-MLE Mean-Value	RMSE	26.448	24.186	420.953	28.130
	MAD	21.790	19.902	346.273	23.222
MFVLE Mean-Value	RMSE	584.253	411.468	17061.371	1131.270
	MAD	403.701	308.844	10830.627	685.123
MPLE Mean-Value	RMSE	27.361	24.897	435.168	28.930
	MAD	22.483	20.496	358.306	23.874

The middle row of Fig 1 shows the MFVLE natural parameter samples with some extreme values replaced, in contrast to the top row, which shows the full set of MCMC-MLE samples. Using winsorization based on the 15th and 85th percentile cutoffs, 294 of the 975 total edge counts (accounting for degeneracy) were replaced when producing the histograms shown in the middle row of Fig 1. While the center MFVLE distributions highlight the regions of highest mass, the majority of samples are spread broadly across the parameter space (e.g., the Edge natural parameter ranges from approximately −1230 to 652, which is unrealistic). The lack of resemblance of the MFVLE mean-value parameter estimates to the true parameters can be somewhat alleviated by replacing extreme values. To look at the medians of the mean-value parameters instead of the means, the MFVLE parameter estimates for *n* = 100 become {394, 341, 3076, 116}. These values lie much closer to the true parameters {393.12, 340.94, 3091.85, 117.74} than the mean-value estimates {910.53, 621.64, 33030.41, 6623.04}. Similarly, the medians of the MFVLE natural parameter estimates {−3.987,2.132,0.000,−0.007} are much closer to true values than the means. As an alternative to the full sample errors in Table 2, the winsorized samples of Fig 1 can be used to allow slight improvements in MFVLE, leading to its natural parameters having RMSE of {2.372, 3.230, 0.051, 0.104} and MAD of {1.759, 2.311, 0.038, 0.088}. While ignoring extreme values makes MFVLE appear more favorable, we note again the large spread of MFVLE samples and its nontrivial tendency to produce outliers (roughly 10% of samples using the 1.5× interquartile range rule of thumb). Moreover, trimming and winsorizing to produce more adequate settings for comparison only result in marginal improvements to RMSE and MAD for MFVLE at the cost of altering a large set of parameter estimates. Even when accounting for MFVLE’s pronounced distributional shortcomings, MCMC-MLE still clearly outperforms it in both the natural and mean-value parameterizations.

**Fig 1:**
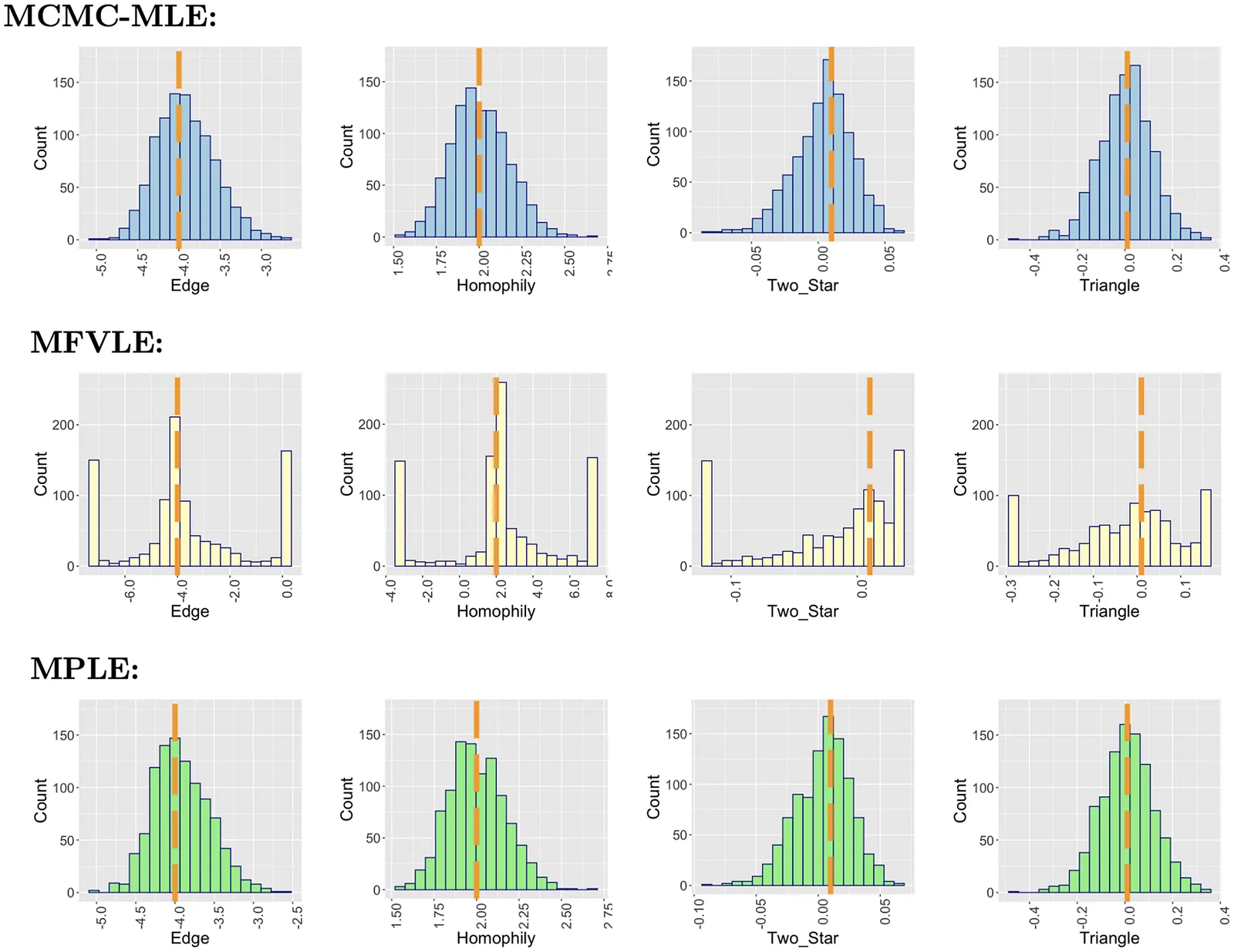
Distribution of MCMC-MLE, MFVLE, and MPLE natural parameters estimates for model using *n* = 100. True values shown by orange line. MFVLE estimates are winsorized at the 15th and 85th percentiles, and use a different scale compared to MCMC-MLE and MPLE.

### 3.3 Performance at larger network sizes

We now consider if larger network sizes improve the performance of the estimators. For very large network sizes the standard MPLE can be computationally burdensome, so we use a variant of it where selective sampling of the dyads is undertaken. Specifically, ties are selected with probability 1 and non-ties are selected with low probability. Then the pseudo-likelihood is approximated by the inverse-probability estimator based on the sample. This is often called “case-control” or “endogenous” sampling and has been available in ergm for two decades. It dramatically speeds up the computation with minimal and controllable loss in precision.

In Table 3 we show results for a large network size (*n* = 2,000). This extends the pattern we see for smaller networks: The MCMC-MLE, MPLE and sampled MPLE are very accurate, while the MFVLE is completely unrealistic due to the high proportion of extreme values and high variance. We also note the relative computational costs of these methods. We see that the MPLE and sampled MPLE are quite fast, while the MCMC-MLE remains computationally feasible at approximately 300 minutes per estimate. The MFVLE is extremely slow, in addition to being inaccurate. Finally, we repeated the study using a very large network (*n* = 10,000). For networks of this size the MFVLE algorithm did not finish after two weeks running. It is not computable for large networks (that is, *n* > 10,000 and not extremely sparse). The MPLE took about 560 minutes per estimate and the sampled MPLE took about 127 minutes per estimate. Both were very accurate. The sampled MPLE could be made much faster by reducing the number of sampled dyads (here set to very large value, 5 million).

**Table 3:** Original study with the initial values for both MCMC-MLE and MFVLE as MPLE. The model has true natural parameters at θ=(−4,2,1/n,1/n) and the values in the table are scaled by (1,1,*n*,*n*). The CPU times are in units of minutes per estimate. All computations were completed on an Intel Xeon Platinum 8160 CPU with 256 GB RAM. The number in parentheses indicates the number of simulated networks fitted for each method. Fewer networks were fitted for MCMC-MLE and MFVLE due to their higher computational cost.

n = 2000	Edge	Homophily	Two-Star	Triangle	CPU time
True Natural parameters	−4.000	2.000	1.000	1.000	
MPLE Sampled Natural (200 simulations)	−4.001	2.000	1.016	0.784	0.32 min
MPLE Natural (200 simulations)	−4.005	2.000	1.043	0.859	1.2 min
MCMC-MLE Natural (10 simulations)	−4.032	2.004	1.218	0.198	300 min
MFVLE Natural (10 simulations)	−3.462	−15.818	−16.4	−25.5	2775 min

In summary, these results show that the apparent accuracy of the MFVLE in their Table 1 does not reflect genuine estimation performance, but instead results from starting the algorithm at the true values. The algorithm does not move from its starting value and hence appears to be accurate. When not started at the truth, MFVLE results appear to be worse than those of MPLE and MCMC-MLE.

From a computational point of view, these experiments also highlight very different scaling behaviors of the various estimators. Each MFVLE fit requires optimization over all dyads at every variational iteration, so its cost grows at least on the order of *O*(*n*^2^) in the number of nodes, and in practice was dominated by the repeated evaluation of dense expectations of the sufficient statistics. By contrast, MCMC-MLE updates a single dyad at a time and the sampled MPLE operates on a subsample of dyads; for the parameter ranges we consider their effective cost grows much more gently with *n*, and is largely driven by the number of sampled dyads or MCMC iterations rather than by the total number of possible ties. The CPU times in Table 3 are therefore not only case-specific numbers, but also indicative of fundamental scaling limitations of the MFVLE relative to standard likelihood-based methods.

### 3.4 How realistic are these models?

Consider an ERGM for which the tie variables are independent. Specifically, where


$$\begin{array}{c} p_{\theta }(Y=y|X=x)=\prod_{ij}p_{\theta }(y_{ij}|X=x)=\frac{\exp(\eta (\theta )\cdot vec(y))}{c(\theta ,G_{n})}\;y\in G_{n} \end{array}$$ (5)

where η(θ) is a known *O*(*n*^2^)-dimensional vector of dyad-specific parameters, parameterized by θ, and vec(y) is the corresponding vectorized version of *y*. For such a model the MPLE is identical to the MLE and can be computed deterministically. Such tie-independent models are flexible, but cannot capture the complex interactions that motivate most network modeling in economics and other social sciences. These models are relevant here because standard methods like the MPLE will tend to be both accurate and computationally efficient for network models that are close to tie-independent.

How dependent is our model? The model contains two network statistics that allow it to deviate from tie-independent models – the Two-star and Triangle statistics. A simple test is to generate networks from models in which the Triangle natural parameter is set to zero, with all other parameters held at their original values. The mean-value parameters of these reduced models can then be compared to the original mean-value parameters (that is, the expected numbers of Two-stars and Triangles).

In Table 4, the ERGM for network size *n* = 100 is adjusted so that only the Triangle natural term is set to zero. This table shows that setting the Triangle parameter to zero causes minimal change in the expected network statistics. For instance, the ratio of the expected number of triangles (117.2 → 113.6) formed to the expected number of total edges (393.0 → 389.4) changes from 0.298 to 0.292. This indicates that the chosen Triangle natural parameter (0.01) is so small that the model is very close to a tie-independent model. Hence the true model parameters are unlikely to be interesting for economic models, nor challenging to estimate as the MPLE will do well in this case.

**Table 4:** Model for *n* = 100 with decreased tie dependence.

θ	−4	2	1/*n*	$\theta_{Triangle}$
$\theta_{Triangle}$	Edge	Homophily	Two-Star	Triangle
0.010	393.043	340.730	3091.731	117.206
0.000	389.376	337.672	3032.674	113.559

From an applied perspective, such parameter settings correspond to networks with very weak clustering relative to what is typically observed in social or economic data. Empirical friendship, collaboration, and trade networks with similar edge densities generally exhibit substantially higher Triangle counts and stronger dependence between dyads than the specifications considered by Mele and Zhu. Thus their calibration places the model in a regime that is simultaneously easy for pseudo-likelihood based methods and unrepresentative of the kinds of networks that motivate the use of ERGMs in practice.

### 3.5 Comparison to more realistic models

This analysis suggests that a more relevant and powerful assessment of the estimation methods would come from networks with increased transitivity, as these are more realistic and interesting theoretically. For simplicity, we take as our measure of transitivity of a model its expected number of triangles, with a model producing higher expected numbers of triangles being more transitive.

As noted in Section 2.1, the model in Mele and Zhu [18] is prone to degeneracy and will be for parameter values chosen close to realistic networks. The parameter values chosen in the original study are close to tie-independent and hence the degeneracy is not strong. However, if we choose parameter values corresponding to increased transitivity, they will be degenerate or nearly so. This behavior is not realistic, except in rare cases where the degeneracy reflects economic theory. One solution is to use other terms that capture the complex dependencies while being less prone to degeneracy [34]. Here we consider the tapered ERGM variant of this model which circumvents the issue of degeneracy while maintaining the desirable features of ERGMs, including the retention of the Triangle term [12]. We adopt the tapered ERGM in the comparison because it supplies a model with realistic transitivity that remains non-degenerate. The tapered version of an ERGM model is itself an ERGM, adding terms that taper the entropy of the original ERGM and reduce unrealistic behavior in models with increased dependence and transitivity. Its coefficients therefore carry the same interpretation as those of the untapered model built from the same sufficient statistics. The tapering terms are chosen to perturb the model only slightly near the target mean-value parameters, so each tapered coefficient stays numerically close to the coefficient of the untapered model targeting the same mean value. Tapered ERGM models have been shown to have these and other good statistical and probabilistic properties (For a full treatment, see Blackburn and Handcock [35]).

Intuitively, tapering modifies the likelihood by down-weighting network configurations with extreme values of the sufficient statistics via an additional term in the model that penalizes extreme variation. This can be viewed as an entropy-regularization device that keeps the distribution away from the extreme faces of the convex support of *Z*(*Y*), where standard ERGMs tend to concentrate when they are close to degenerate. Thus tapering improves the stability and robustness of the models. This stability and robustness extends to MCMC-based inference for them. MCMC-MLE for tapered ERGM is implemented by the R package ergm.tapered on GitHub [36], and the tapering term is applied to the likelihood function in R package mfergm for MFVLE.

To consider models that are close to the original ERGM, but with higher transitivity, we can choose tapered ERGM models with similar mean-value parameters, but with increased transitivity. Specifically, we choose models with 50% more transitivity than the original version of the model, thus factoring the original expected count for triangles by 1.5. This is done by solving equation (2) for θ with the left hand side set to the desired mean-value parameter. This results in natural parameter values for the Triangle term that are substantially above zero. Without tapering, which helps move sampled statistics away from degenerate regions, substantial increases in transitivity are typically infeasible due to model degeneracy. This is particularly true for our model specification, which includes the raw Triangle statistic.

### 3.6 Increased transitivity model

Table 5 is a version of Table 1 with the comparison of the estimation methods with the Triangle mean-value increased by approximately 50%, while holding all other terms approximately constant. In the table, we can see this change reflected in the comparison between original mean-values and increased-transitivity mean-values. Taking a large sample with specified target statistics allows for the desired mean-value parameters – and thus the one-to-one mapped natural parameters – to be computed. For *n* = 100 we see the Triangle natural parameter is positive (0.60) to a greater magnitude compared to the original value (0.01). The mean-value parameter reflects the approximately 50% increase in transitivity (117.5→176.1) introduced in this section.

**Table 5:** Tapered version of model for *n* = {50, 100} with increased transitivity, where the mean-value parameter for Triangle term is increased by a factor of γ≈1.5.

n = 50	Edge	Homophily	Two-Star	Triangle	Degeneracy
Increased-Transitivity Tapered θ	−3.732	1.795	−0.033	0.630	–
MCMC-MLE Natural	−3.674	1.836	−0.044	0.594	0.0%
MFVLE Natural	−17.429	−6.176	−0.463	0.224	10.6%
MPLE Natural	−3.614	1.827	−0.048	0.599	0.0%
Original Mean-Value	95.75	82.71	363.42	13.91	–
Increased-Transitivity Mean-Value	95.31	82.66	363.21	20.87	–
MCMC-MLE Mean-Value	95.39	82.76	362.81	20.72	–
MFVLE Mean-Value	80.44	55.37	369.93	14.61	–
MPLE Mean-Value	95.93	83.13	365.07	20.77	–
**n = 100**	**Edge**	**Homophily**	**Two-Star**	**Triangle**	**Degeneracy**
Increased-Transitivity Tapered θ	−3.022	1.530	−0.066	0.600	–
MCMC-MLE Natural	−3.009	1.552	−0.068	0.589	0.0%
MFVLE Natural	−15.194	−1.217	−0.245	0.401	7.2%
MPLE Natural	−2.985	1.548	−0.068	0.590	0.0%
Original Mean-Value	393.05	341.02	3092.06	117.48	–
Increased-Transitivity Mean-Value	393.45	341.16	3092.28	176.12	–
MCMC-MLE Mean-Value	393.53	341.20	3093.09	175.92	–
MFVLE Mean-Value	277.97	201.90	2720.45	141.20	–
MPLE Mean-Value	393.85	341.36	3096.08	176.01	–

The results in Table 5 show that MCMC-MLE provides greater accuracy than MFVLE, and Table 6 further demonstrates that the MCMC-MLE attains superior computational efficiency. For *n* = 50, the MFVLE estimate of the natural parameter for the Triangle term is 0.224 compared to the true value of 0.630, while the MCMC-MLE is 0.594. The magnitude of this difference is clarified by comparing the mean-value parameter estimates. The MCMC-MLE mean-value estimate is 20.72 compared to the true value of 20.87, while the MFVLE estimate is 14.61. The MFVLE estimate is unable to capture even the qualitative character of the transitivity, while the MCMC-MLE is very accurate, as would be expected from theory.

**Table 6:** Summary measurements RMSE and MAD for *n* = 100 increased transitivity results in Table 5.

θ={−3.022,1.530,−0.066,0.600}		Edge	Homophily	Two-Star	Triangle
MCMC-MLE Natural	RMSE	0.511	0.282	0.059	0.217
	MAD	0.433	0.244	0.051	0.185
MFVLE Natural	RMSE	9.951	15.428	0.185	0.393
	MAD	9.368	14.580	0.176	0.370
MPLE Natural	RMSE	0.523	0.283	0.061	0.222
	MAD	0.443	0.244	0.052	0.191
MCMC-MLE Mean-Value	RMSE	3.819	5.084	22.463	4.128
	MAD	3.364	4.331	19.076	3.513
MFVLE Mean-Value	RMSE	47.300	54.517	206.291	14.563
	MAD	34.567	40.567	157.697	11.892
MPLE Mean-Value	RMSE	3.845	5.138	22.517	4.158
	MAD	3.388	4.389	19.160	3.545

Table 6 shows that the tapering effect has reduced all RMSE and MAD values compared to Table 2 despite the increased transitivity. We see that the MCMC-MLE and MPLE remain accurate and stable estimators for this model with the RMSE and MAD slightly better for the MCMC-MLE than the MPLE. The strong performance of MPLE here may look surprising, since MPLE is known to degrade as tie-dependence increases. This happens because tapering keeps the sampled statistics away from the degenerate region of the sample space, which is where the pseudo-likelihood approximation breaks down. By holding the model away from that boundary, tapering weakens the dependence that normally inflates MPLE bias, and MPLE recovers close to the accuracy of MCMC-MLE even at this higher level of transitivity. However, the MFVLE performs very poorly. To start, the MFVLE estimate fails to converge in 10.6% of the fits (despite the starting value being very accurate) whereas tapering has allowed MCMC-MLE to always produce a result. Compared to the original study, where MFVLE had a 9% degeneracy in Table 1, the slight increase observed here can be explained by two opposing effects. Increased transitivity pushes MFVLE toward more degenerate configurations, whereas the tapering effect pulls the model away from degeneracy. While setting a stronger tapering parameter would lead to less degeneracy for MFVLE, the results produced here (using default tapering parameter of 2, detailed in Blackburn and Handcock [35]) highlight its behavior. Secondly, both the natural and mean-value parameters are biased. Third, the RMSE and MAD are quite poor compared to those of MCMC-MLE and MPLE. For the mean-value Triangle parameter, MFVLE shows an RMSE of 14.56 where MCMC-MLE has RMSE of 4.128 and MPLE has RMSE 4.158. We have separately computed MFVLE without tapering, and those natural parameters yielded RMSE of {17.023, 22.456, 0.144, 0.199} and MAD of {15.414, 20.708, 0.128, 0.183}. Both of these measures are noticeably worse than those for its tapered counterpart. As a strict comparison between the variational methods, MFVLE is dominated by the MPLE on all measures including computation time.

In our Section 3 findings, we see that the number of triangles formed under the models used by Mele and Zhu [18] does not differ significantly from that under the same model with the Triangle term set to zero. Under a model with no transitivity, MPLE produces MLE results and therefore leads to good mean-field approximation results due to initialization. Furthermore, the increase in transitivity for the ERGM led to the mean-field method producing dramatically fewer edges compared to true simulated networks.

## 4. Discussion

The fitting of a complex model that realistically represents observed social and economic network data is challenging. The dependence structure that makes these models appealing also makes likelihood-based inference computationally demanding. As such, the search for accurate and computationally tractable methods is ongoing. While likelihood-based estimation methods can be computationally expensive, they do have a strong track record of success and a long history of development.

In this paper we have shown that there is little empirical and conceptual support for the mean-field variational estimator. We show that there is a design flaw in the original simulation study [18] that invalidates their empirical results. We also argue that, even if those results had been correct, they would not have provided much support for the method as the models they were assessed on are unrealistically close to tie-independent models. When we alter the model to make it more realistic by inducing stronger dependence among ties, the estimator performs poorly and is more computationally expensive compared to both MCMC-MLE and the cheap sampled MPLE.

Variational methods do have value when they are applied to appropriate model classes with parameter values in regions where the approximation is not bad. This is challenging for ERGMs, which constitute a highly flexible class of models: depending on the choice of sufficient statistics, they can induce strong and complex dependence among ties that is difficult for variational approximations to capture. In addition, variants that aim to capture realistic and complex network structure tend to be those where the approximation does poorly. Our results suggest that mean-field variational approximation should not be used as a default method for ERGMs, and a more robust strategy would be to use MPLE for initialization and MCMC-MLE for final inference.

Estimates based on variational approximations can also underestimate the estimation uncertainty. Tan and Friel [37] show that in Bayesian inference for ERGM, variational approximations can underestimate the posterior variances.

Our analysis also illustrates the value of tapered ERGM both for model specification and estimation. These models retain the expressiveness of a traditional ERGM while allowing practical MCMC-based methods of inference. In sum, when using ERGM, researchers should usually prefer the tapered ERGM. For inference, they should use MPLE for initialization and MCMC-MLE, if computationally feasible, for reliable inference. MFVLE should not be used as a default estimator for ERGMs with substantively meaningful endogenous dependence, and in the settings studied here it is dominated by MPLE or MCMC-MLE.

The computations in this paper were performed using the ergm [9] and ergm.tapered [35,36] R-packages within the statnet suite [2]. This is sophisticated, accessible, user-friendly and open-source software for fitting and simulating ERGM. The R code to reproduce the results of this paper using these packages is available on GitHub [38] and published on Zenodo [39].

For completeness it is worth emphasizing that the fully factorized mean-field family studied by Mele and Zhu is only one member of a broader class of deterministic approximations to ERGMs. Composite or pseudo-likelihood based methods, corrected pseudo-likelihoods [14], and variational approximations based on richer dependence structures (for example, Bethe approximations [27] and loopy belief propagation [40]) retain more of the local dependence among ties than a product-Bernoulli mean-field family. Our negative results therefore should not be interpreted as a blanket indictment of all deterministic or variational approaches for ERGMs, but rather as evidence that a tie-independent mean-field family is too restrictive to serve as a default likelihood approximation for models with substantively meaningful transitivity and clustering.

## References

[1] Barndorff-Nielsen OE. Information and exponential families in statistical theory. New York: Wiley; 1978.

[2] Handcock MS, Hunter DR, Butts CT, Goodreau SM, Morris M. statnet: Software tools for the representation, visualization, analysis and simulation of network data. J Stat Softw. 2008;24(1):1548–7660. doi: 10.18637/jss.v024.i01 18618019 PMC2447931

[3] Durlauf SN, Ioannides YM. Social interactions. Annu Rev Econ. 2010;2:451–78. doi: 10.1146/annurev.economics.050708.143312

[4] Krivitsky PN, Handcock MS. A separable model for dynamic networks. J R Stat Soc B Stat Methodol. 2014;76(1):29–46. doi: 10.1111/rssb.12014 24443639 PMC3891677

[5] Boucher V, Mourifié I. My friend far, far away: A random field approach to exponential random graph models. Econom J. 2017;20(3):S14–46. doi: 10.1111/ectj.12096 31007564 PMC6459320

[6] De Paula A. Econometrics of network models. Advances in Economics and Econometrics: Eleventh World Congress. Cambridge: Cambridge University Press; 2017. p. 268–323.

[7] van der Pol J. Introduction to network modeling using exponential random graph models (ERGM): Theory and an application using R. Comput Econ. 2019;54:845–75. doi: 10.1007/s10614-018-9853-2

[8] Frank O, Strauss D. Markov graphs. J Am Stat Assoc. 1986;81:832–42.

[9] Handcock MS, Hunter DR, Butts CT, Goodreau SM, Morris M. ergm: Fit, simulate and analyze exponential-family models for networks. R package version 4.3; 2022. Available from: https://statnet.org 10.18637/jss.v024.i03 PMC2743438 19756229

[10] Krivitsky PN. Exponential-family random graph models for valued networks. Electron J Stat. 2012;6:1100–28. doi: 10.1214/12-EJS696 24678374 PMC3964598

[11] Morris M, Handcock MS, Hunter DR. Specification of exponential-family random graph models: Terms and computational aspects. J Stat Softw. 2008;24(4):1548–7660. doi: 10.18637/jss.v024.i04 18650964 PMC2481518

[12] Fellows I, Handcock MS. Removing phase transitions from Gibbs measures. Proc. of AISTATS; 2017. p. 289–97.

[13] Geyer CJ, Thompson EA. Constrained Monte Carlo maximum likelihood calculations (with discussion). J R Stat Soc B. 1992;54:657–99.

[14] van Duijn MAJ, Gile KJ, Handcock MS. A framework for the comparison of maximum pseudo likelihood and maximum likelihood estimation of exponential family random graph models. Soc Netw. 2009;31(1):52–62. doi: 10.1016/j.socnet.2008.10.003 23170041 PMC3500576

[15] Hunter DR, Handcock MS, Butts CT, Goodreau SM, Morris M. ergm: A package to fit, simulate and diagnose exponential-family models for networks. J Stat Softw. 2008;24(3):nihpa54860. doi: 10.18637/jss.v024.i03 19756229 PMC2743438

[16] Hummel RM, Hunter DR, Handcock MS. Improving simulation-based algorithms for fitting ERGMs. J Comput Graph Stat. 2012;21(4):920–39. doi: 10.1080/10618600.2012.679224 26120266 PMC4479216

[17] Krivitsky PN, Hunter DR, Morris M, Klumb C. ergm 4: New features. arXiv:2106.04997 [Preprint]. 2021. doi: 10.48550/ARXIV.2106.04997

[18] Mele A, Zhu L. Approximate variational estimation for a model of network formation. Rev Econ Stat. 2023;105(1):113–24. doi: 10.1162/rest_a_01023

[19] Handcock MS. Assessing degeneracy in statistical models of social networks. Center for Statistics and the Social Sciences, University of Washington. Working Paper No. 39; 2003. Available from: https://csss.uw.edu/Papers/wp39.pdf

[20] Handcock MS. Statistical models for social networks: inference and degeneracy. In: Breiger R, Carley K, Pattison P, editors. Dynamic social network modeling and analysis. Washington (DC): National Academies Press; 2003. p. 229–52.

[21] Schweinberger M. Instability, sensitivity, and degeneracy of discrete exponential families. J Am Stat Assoc. 2011;106(496):1361–70. doi: 10.1198/jasa.2011.tm10747 22844170 PMC3405854

[22] Schweinberger M, Krivitsky PN, Butts CT, Stewart JR. Exponential-family models of random graphs: Inference in finite, super and infinite population scenarios. Stat Sci. 2020;35:627–62. doi: 10.1214/19-STS743

[23] Chatterjee S, Diaconis P. Estimating and understanding exponential random graph models. Ann Stat. 2013;41(5):2428–61. doi: 10.1214/13-AOS1155

[24] Bickel PJ, Chen A, Levina E. The method of moments and degree distributions for network models. Ann Stat. 2011;39(5). doi: 10.1214/11-aos904

[25] Ellis RS, Newman CM. Limit theorems for sums of dependent random variables occurring in statistical mechanics. Z Wahrsch Verw Gebiete. 1978;44(2):117–39. doi: 10.1007/bf00533049

[26] Dembo A, Montanari A. Ising models on locally tree-like graphs. Ann Appl Probab. 2010;20(2). doi: 10.1214/09-aap627

[27] Ricci-Tersenghi F. The Bethe approximation for solving the inverse Ising problem: A comparison with other inference methods. J Stat Mech. 2012;2012(08):P08015. doi: 10.1088/1742-5468/2012/08/p08015

[28] Lokhov AY, Vuffray M, Misra S, Chertkov M. Optimal structure and parameter learning of Ising models. Sci Adv. 2018;4(3):e1700791. doi: 10.1126/sciadv.1700791 29556527 PMC5856491

[29] Biondini G, Moro A, Prinari B, Senkevich O. p-star models, mean-field random networks, and the heat hierarchy. Phys Rev E. 2022;105(1–1):014306. doi: 10.1103/PhysRevE.105.014306 35193208

[30] Krivitsky PN, Handcock MS, Morris M. Adjusting for network size and composition effects in exponential-family random graph models. Stat Methodol. 2011;8(4):319–39. doi: 10.1016/j.stamet.2011.01.005 21691424 PMC3117581

[31] R Core Team. R: A Language and Environment for Statistical Computing; 2012. Available from: http://www.R-project.org/

[32] Snijders TA. Markov chain Monte Carlo estimation of exponential random graph models. J Soc Struct. 2002;3:1–40.

[33] Mele A. mfergm: A package for variational approximations of (some) ERGM models; 2019. Available from: https://github.com/meleangelo/mfergm

[34] Snijders TA, Pattison PE, Robins GL, Handcock MS. New specifications for exponential random graph models. Sociol Methodol. 2006;36:99–153.

[35] Blackburn B, Handcock MS. Practical network modeling via tapered exponential-family random graph models. J Comput Graph Stat. 2023;32(2):388–401. doi: 10.1080/10618600.2022.2116444 37608920 PMC10441622

[36] Handcock MS, Krivitsky PN, Fellows I. ergm.tapered: Tapered Exponential-family models for networks. R package version 1.2; 2021.

[37] Tan LSL, Friel N. Bayesian variational inference for exponential random graph models. J Comput Graph Stat. 2020;29(4):910–28. doi: 10.1080/10618600.2020.1740714

[38] Resch J. Likelihood inference comparisons for exponential random graph models; 2024. Available from: https://github.com/josephresch/VariationalEstimationERGM

[39] Resch J, Handcock MS. Assessing variational likelihood estimation in network formation models: Code repository; 2026. Available from: doi: 10.5281/zenodo.22313605 PMC13641714 42837405

[40] Jain V, Koehler F, Mossel E. The mean-field approximation: Information inequalities, algorithms, and complexity. In: Bubeck S, Perchet V, Rigollet P, editors. Proceedings of the 31st Conference On Learning Theory. vol. 75 of Proceedings of Machine Learning Research. PMLR; 2018. p. 1326–47. Available from: https://proceedings.mlr.press/v75/jain18b.html

